# Identical genetic influences underpin behavior problems in adolescence and basic traits of personality

**DOI:** 10.1111/jcpp.12156

**Published:** 2014-10-25

**Authors:** Gary J Lewis, Claire M A Haworth, Robert Plomin

**Affiliations:** 1 Department of Psychology, University of YorkYork, UK; 2 Department of Psychology, University of WarwickCoventry, UK; 3 MRC Social, Genetic and Developmental Psychiatry Centre, Institute of Psychiatry, King's College LondonLondon, UK

**Keywords:** Behavioral problems, strengths and difficulties, personality, genetics, twins

## Abstract

**Background:**

Understanding the etiology of adolescent problem behavior has been of enduring interest. Only relatively recently, however, has this issue been examined within a normal personality trait framework. Research suggests that problem behaviors in adolescence and beyond may be adequately explained by the taxonomy provided by the basic dimensions of normal personality: Such problem behaviors are suggested to be extreme points on a distribution of the full range of the underlying traits. We extend work in this field examining the extent to which genetic factors underlying the five-factor model of personality are common with genetic influences on adolescent behavior problems (namely, anxiety, peer problems, conduct, hyperactivity, and low prosociality).

**Method:**

A nationally representative twin sample (Twins Early Development Study) from the general population of England and Wales, including 2031 pairs of twins aged 16 years old, was used to decompose variation into genetic and environmental components. Behavioral problems in adolescence were assessed by self-report with the Strengths and Difficulties Questionnaire.

**Results:**

Adolescent behavior problems were moderately associated with normal personality: Specifically, a fifth to a third of phenotypic variance in problem behaviors was accounted for by five-factor model personality traits. Of central importance here, genetic influences underpinning personality were entirely overlapping with those genetic factors underlying adolescent behavior problems.

**Conclusions:**

These findings suggest that adolescent behavior problems can be understood, at least in part, within a model of normal personality trait variation, with the genetic bases of these behavior problems the same as those genetic influences underpinning normal personality.

Read the Commentary for this article on doi: 10.1111/jcpp.12292

## Introduction

Understanding the etiology of adolescent behavioral problems – such as problems with peer relations, hyperactivity, conduct, anxiety, and (low) prosociality – is of importance as such behaviors show links to broad-based life and psychosocial outcomes (Rutter, 1995). While psychopathologies are often viewed from a qualitative perspective (i.e., absence vs. presentation of a problem behavior: American Psychiatric Association, 2000), recent work (Krueger et al., 2011; Widiger & Trull, 2007) has sought to account for individual differences in such behaviors within a model of normal personality traits, such that problem behaviors reflect extreme points on the full range of the respective personality traits. This debate is of substantial contemporary importance in light of the proposition to conceptualize personality disorders from a dimensional perspective for the fifth edition of Diagnostic and Statistical Manual of Mental Disorders (DSM-5) (Krueger et al., 2011). With this in mind, here we sought to examine whether genetic variation on the Strengths and Difficulties Questionnaire – a widely used measure tapping behavior problems in children and adolescents – could be explained within the framework of the major trait perspective of personality, the five-factor model (Costa & McCrae, 1992; Digman, 1990). This approach is in line with much research in adolescent psychopathology suggesting that the dimensional taxonomy posited to underpin adult psychopathology is also a valid reflection of developmental behavioral problems (e.g., Hudziak, Achenbach, Althoff, & Pine, 2007; Tackett, 2006).

### Adolescent behavioral problems

Research has suggested that around one in five adolescents satisfy diagnostic criteria for at least one psychiatric disorder (Merikangas et al., 2010), with specific behavior problems such as conduct disorder, hyperactivity disorder, and anxiety/emotional problems all relatively common among adolescents (Ford, Goodman, & Meltzer, 2003; Merikangas et al., 2010). These behavioral problems are of clear and broad interest because of their links to mental health, school achievement, antisocial behavior, and delinquency (Aunola, Stattin, & Nurmi, 2000; Fergusson, Horwood, & Ridder, 2005; Rutter, 1995).

Several measurement instruments exist to screen for such behaviors (Achenbach, 1991a,b,c; Elander & Rutter, 1996), including – as well as the focus of the current study – the Strengths and Difficulties Questionnaire (SDQ) (Goodman, 1997). The SDQ is a brief dimensional measure that covers the core domains of child and adolescent psychopathology (conduct problems, hyperactivity-inattention, emotional symptoms/anxiety, and peer relations problems) alongside personal strengths (prosociality).

### Etiologies of adolescent behavioral problems: the role of dimensional personality models and genetics

The assessment and taxonomy of normal personality has also been of longstanding interest (John, Naumann, & Soto, 2008). In recent years, broad consensus has emerged concerning the taxonomy of core personality dimensions, with research providing strong support for the existence of five core personality dimensions, commonly referred to as the five-factor model (FFM) (Costa & McCrae, 1992; Digman, 1990). The FFM consists of extraversion (the tendency to experience positive emotions and sociability), neuroticism (the tendency to experience negative affect), conscientiousness (the ability to plan, organize, and complete tasks), agreeableness (the tendency to trust others and show sympathy and altruism), and openness (the tendency to engage in novel activities and emotions). Across several studies, moderate-to-large (typically ≥.40) heritable influences on FFM personality traits have been noted (e.g., Jang, Livesley, & Vernon, 1996; Loehlin, McCrae, Costa, & John, 1998).

Although psychopathology is often conceptualized as qualitatively distinct phenomena (Hudziak et al., 2007; Krueger et al., 2011; Widiger & Trull, 2007), much work suggests that such behaviors exist as the extreme point on a normal personality distribution (Kotov, Gamez, Schmidt, & Watson, 2010; Plomin, Haworth, & Davis, 2009; Saulsman & Page, 2004). This dimensional perspective has also been strongly supported in developmental psychopathology research (cf. Hudziak et al., 2007). With this in mind, researchers have attempted to place adolescent psychological and behavioral functioning, as well as misfunctioning, within the five-factor framework. For example, in a large sample of Russian adolescents, FFM traits explained 11–25% of the variance on four of the five subscales of the SDQ (data on hyperactivity was not reported) (Slobodskaya, 2007). Similar results were reported in a sample of Dutch adolescent boys and girls (Muris, Meesters, & Diederen, 2005). More broadly, a range of studies have noted links between personality traits and childhood and adolescent behavioral problems. For example, measures of impulsivity and sensation seeking have been significantly associated with antisocial behaviors across childhood (Raine, Reynolds, Venables, Mednick, & Farrington, 1998; Tremblay, Pihl, Vitaro, & Dobkin, 1994) and adolescence (Lynam et al., 2000). Aspects of attention-deficit/hyperactivity disorder have been also been linked to personality: Symptoms of inattention (IA) and hyperactivity-impulsiveness (HI) have both been associated with high impulsivity, with IA also being associated with higher punishment sensitivity, and HI being associated with higher reward sensitivity (Gomez & Corr, 2010).

Research examining the etiology of such problem behaviors has also indicated a role for genetics. For instance, childrens' and adolescents' scores (ages 5–17) on the conduct problems scale of the SDQ have been reported to be substantially heritable (35%–77%) (Scourfield, Van den Bree, Martin, & McGuffin, 2004). Similarly, high heritabilities were found for all of the SDQ scales at age 7 (Saudino, Ronald, & Plomin, 2005). Moderate to substantial heritabilities (≥.40) have also been found for related measures, such as antisocial behavior and callous-unemotional traits in adolescence (Viding, Blair, Moffitt, & Plomin, 2005), and internalizing and externalizing behaviors (Bartels et al., 2004). In sum, then, child and adolescent behavior problems across multiple domains exhibit moderate-to-large heritable effects.

### The current study

These findings, noting that personality and psychopathology are individually heritable, as well as phenotypically associated, give rise to the question of whether these phenotypic links are mediated by genetic and environmental factors (Krueger & Markon, 2006). Work in parallel fields has explored this issue. For instance, genetic influences underlying neuroticism have been shown to account for between one-third and one-half of the genetic risk for internalizing disorders in adults (Hettema, Neale, Myers, Prescott, & Kendler, 2006; Kendler & Gardner, 2011; Mackintosh, Gatz, Wetherell, & Pedersen, 2006). Similarly, neuroticism has shown a substantial genetic correlation with major depression (Fanous, Gardner, Prescott, Cancro, & Kendler, 2002). In youth populations, research has also demonstrated links between behavioral problems and core aspects of personality. For example, negative affect has been shown to share genetic influences with both internalizing and externalizing disorders in childhood (Mikolajewski, Allan, Hart, Lonigan, & Taylor, 2013). Negative emotionality has been reported to show genetic links with both conduct disorder and depressive disorder (Tackett, Waldman, Van Hulle, & Lahey, 2011). Finally, conduct disorder has been found to contain shared genetic influences with three socioemotional traits: prosociality, negative emotionality, and daring (Waldman et al., 2011). Limited work to date, however, has examined whether genetic factors underlying behavioral problems in adolescence can be understood within the specific rubric of FFM personality traits, as well as addressing adolescent behavioral functioning using a broad-based measurement tool (such as the SDQ). To address this gap in the literature, here we sought to examine the extent to which genetic factors underpinning SDQ constructs can be understood within the genetic bases underlying FFM traits using a large nationally representative sample of English and Welsh adolescent twins.

## Methods

### Sample

The current study sample was drawn from the age 16 assessment wave in the Twins Early Development Study (TEDS), which is an ongoing longitudinal study following monozygotic (MZ) and dizygotic (DZ) twins born in England and Wales between 1994 and 1996 (Haworth, Davis, & Plomin, 2013). The TEDS sample is representative of the UK population (Kovas, Haworth, Dale, & & Plomin, 2007). Ethical approval was provided by the King's College London ethics committee (reference: 05/Q0706/228), and the parents of the twins provided informed written consent. Zygosity was assessed through a parent questionnaire of physical similarity, which has been shown to be over 95% accurate when compared to DNA testing (Price et al., 2000). For cases where zygosity was unclear from this questionnaire, DNA testing was conducted. The current sample reflected a subset of the broader cohort and consisted of the following number of twin pairs: MZ male pairs: *n* = 282 (FFM) and 635 (SDQ); MZ female pairs: *n* = 501 (FFM) and *n* = 912 (SDQ); DZ male pairs: *n* = 267 (FFM) and *n* = 580 (SDQ); DZ female pairs: *n* = 396 (FFM) and *n* = 811 (SDQ); and DZ opposite-sex pairs: *n* = 566 (FFM) and *n* = 1340 (SDQ).

### Measurements

The FFM questionnaire was completed online as part of a TEDS assessment wave at age 16. The SDQ measures were sent by mail to the twins for home completion (along with other questionnaires) approximately 6 months later.

#### Strength and Difficulties Questionnaire (SDQ)

The Strengths and Difficulties Questionnaire (SDQ) is a short (25 items) and a reliable instrument (Goodman, 2001; Stone, Otten, Engels, Vermulst, & Janssens, 2010) for measuring psychosocial problems in children and adolescents (Goodman, 1997). The self-report version of the SDQ (used here) was intended for use for children and adolescents from age 11 to 16 years (Goodman, Meltzer, & Bailey, 1998). The SDQ consists of five scales measuring anxiety symptoms, conduct problems, hyperactivity-inattention, peer problems, and prosocial behavior. Importantly, the SDQ is an efficient screening tool for child and adolescence psychopathology (Goodman, Ford, Simmons, Gatward, & Meltzer, 2000). Descriptive statistics of the SDQ measures from the current sample are included in Table1. Cronbach's alpha was acceptable for most of the measures, although conduct and peer problems were lower (α = .53 and .56, respectively). It is worth noting, however, that some debate exists over whether modest Cronbach's alpha values indicate need for concern. Indeed, if one uses a broad content coverage and quickly administrable instrument (i.e., few items per scale), as in the current study, one should expect, and perhaps even desire, such ‘modest’ alphas (e.g., Boyle, 1991).

**Table 1:** Descriptive statistics for FFM traits and SDQ subscales

Measure	α	Means (*SD*)							ANOVA			
		All	MZm	MZf	DZm	DZf	DZosm	DZosf	Sex	Zyg	Sex × Zyg	*R*^2^
Personality												
Neuroticism	.68	2.58 (.67)	2.43 (.61)	2.65 (.72)	2.49 (.67)	2.68 (.63)	2.49 (.68)	2.61 (.66)	40.11**	1.30	1.02	.02
Extraversion	.70	3.66 (.62)	3.67 (.62)	3.66 (.62)	3.63 (.63)	3.68 (.60)	3.56 (.62)	3.73 (.64)	2.78	.28	4.65*	<.01
Openness	.63	3.58 (.59)	3.58 (.63)	3.58 (.58)	3.55 (.61)	3.58 (.59)	3.55 (.59)	3.61 (.58)	.62	.03	.62	<.01
Agreeableness	.68	3.67 (.59)	3.57 (.58)	3.78 (.59)	3.51 (.58)	3.74 (.60)	3.56 (.56)	3.75 (.59)	66.35**	1.56	.03	.03
Conscientiousness	.78	3.73 (.62)	3.68 (.62)	3.83 (.60)	3.66 (.61)	3.75 (.65)	3.61 (.61)	3.74 (.61)	23.35**	5.95*	.58	.01
SDQ												
Anxiety	.66	2.73 (2.22)	1.81 (1.81)	3.35 (2.35)	2.03 (1.95)	3.35 (2.24)	1.97 (1.77)	3.41 (2.30)	467.85**	2.44	1.61	.10
Conduct	.53	1.63 (1.47)	1.64 (1.47)	1.47 (1.36)	1.78 (1.44)	1.61 (1.51)	1.76 (1.52)	1.63 (1.48)	11.38**	8.49**	.48	.01
Hyperactivity	.74	3.53 (2.33)	3.36 (2.29)	3.31 (2.27)	3.57 (2.39)	3.66 (2.41)	3.86 (2.26)	3.50 (2.34)	1.55	18.03**	.36	.01
Peer problems	.56	1.56 (1.52)	1.53 (1.52)	1.51 (1.51)	1.72 (1.63)	1.51 (1.42)	1.66 (1.53)	1.50 (1.54)	4.56*	2.51	2.39	<.01
Prosociality	.70	7.11 (1.97)	6.55 (2.01)	7.60 (1.83)	6.49 (1.95)	7.58 (1.84)	6.43 (1.94)	7.68 (1.79)	334.53**	.32	.96	.08

One randomly chosen member from each twin pair; the total sample size is 2270–2278 for the personality scales and 4318–4320 for the SDQ scales. *M* = mean (*SD* = standard deviation). α = Cronbach's alpha for scale scores collapsed across sex and zygosity. MZ, monozygotic; DZ, dizygotic; m, male; f, female; os, opposite sex; ANOVA results = F statistic.

* *p* < .05

** *p* < .01.

#### Five-factor model rating form

The five-factor model (FFM) rating form (Mullins-Sweatt, Jamerson, Samuel, Olson, & Widiger, 2006) is a 30-item inventory with six items for each of the five dimensions of normal personality: neuroticism, extraversion, openness, agreeableness, and conscientiousness. Each item was rated on a 1–5 scale from ‘extremely low’ to ‘extremely high’. Descriptive statistics for the FFM measures from the current sample are included in Table1. Cronbach's alpha was acceptable for each of the measures. Test–retest reliability was high for each of the FFM measures: 23 twin pairs completed a paper-and-pencil version of the measures approximately 2 months after the first assessment, with zero-order correlations between measurement occasions ranging from .61 (openness) to .78 (neuroticism, agreeableness, and conscientiousness).

### Data analysis

Correlations between twins differing in their degrees of genetic relatedness (e.g., MZ and DZ twins) are informative as a guiding heuristic to relative magnitudes of genetic and environmental effects (Plomin, DeFries, Knopik, & Neiderhiser, 2013). The presence of genetic effects is inferred if correlations between MZ twins are larger than correlations for DZ twins. The presence of shared environmental effects is inferred if correlations for DZ twins are larger than half the magnitude of the correlations for MZ twins. Finally, nonshared environmental effects are inferred if correlations for MZ twins are less than 1.0, and so this variance component also contains measurement error. These correlation analyses were extended using formal model fitting of variance–covariance matrices for the twin data. This approach allows parameter estimates in univariate models to be formally tested for significance as well as allowing multivariate models – including sex-limitation models – to be analyzed.

## Results

Descriptive statistics for all measures for one individual in a twin pair selected at random are detailed in Table1. Means and variances for the majority of the variables (Table1) differed significantly between zygosity and sex. However, with the exception of anxiety and prosociality (girls had higher means), these differences were modest in magnitude (<3% of the variance), with the large sample size allowing even small effects to reach significance.

### Phenotypic associations between SDQ and FFM traits

Zero-order correlations for FFM traits and SDQ measures are detailed in Table2. To determine independent effects of FFM traits on SDQ scores we performed a series of linear multiple regression analyses with each of the SDQ subscales regressed on FFM traits (see Table3). FFM traits explained from 30% of the variance for anxiety to 17% of the variance for both conduct and hyperactivity. Anxiety was significantly associated with agreeableness (low) extraversion, openness, and neuroticism. Conduct was significantly associated with (low) agreeableness, neuroticism, extraversion, and (low) conscientiousness. Hyperactivity was significantly associated with neuroticism, extraversion, openness, and (low) conscientiousness. Peer problems were significantly associated with (low) agreeableness, (low) extraversion, neuroticism, and openness. Finally, prosociality was significantly associated with agreeableness, extraversion, and conscientiousness.

**Table 2:** Phenotypic correlations between FFM traits and SDQ measures

	N	E	O	A	C	Anx.	Cond.	Hyp.	Peer
E	**−.36**								
O	**−**.03	**.25**							
A	**−.16**	**.18**	**.23**						
C	**−.17**	**.21**	**.08**	**.29**					
Anx.	**.51**	**−.26**	.06*	.06*	**−**.06*				
Cond.	**.29**	.04	**−**.01	**−.26**	**−.23**	**.22**			
Hyp.	**.25**	.01	.05	**−.13**	**−.33**	**.30**	**.46**		
Peer	**.33**	**−.34**	.01	**−.13**	**−.09**	**.34**	**.22**	**.17**	
Prosoc.	**−.13**	**.29**	**.13**	**.34**	**.25**	**.06**	**−.23**	**−.19**	**−.22**

Bolded coefficients = *p* < .001. One randomly chosen member from each twin pair; *n* = 2276–2285 (within FFM traits), *n* = 4305–4308 (within SDQ measures), *n* = 1700–1708 (across FFM traits and SDQ measures). A, Agreeableness; C, Conscientiousness; E, Extraversion; N, Neuroticism; O, Openness; Anx., anxiety; Cond., conduct; Hyp., hyperactivity; Peer, peer problems; Prosoc., prosociality.

* *p* < .05.

**Table 3:** Linear regressions (with standardized beta coefficients) of each of the five SDQ subscales on the FFM traits

	Anxiety	Conduct	Hyperactivity	Peer problems	Prosociality
N	**.48**	**.27**	**.22**	**.20**	**−**.02
E	**−.15**	**.19**	**.12**	**−.31**	**.22**
O	.05*	.02	.08**	**.09**	.01
A	**.20**	**−.24**	**−**.01	**−**.08**	**.28**
C	.04	**−.12**	**−.34**	.04	**.13**
F	**145.03**	**66.88**	**69.35**	**75.03**	**85.04**
R^2^	.30	.17	.17	.18	.20
Adj. R^2^	.30	.17	.17	.18	.20

Bolded coefficients = *p* < .001. One randomly chosen member from each twin pair; *n* = 1673–1674. Adj. R2, Adjusted R2; A, Agreeableness; C, Conscientiousness; E, Extraversion; N, Neuroticism; O, Openness.

* *p* < .05

** *p* < .01.

### Univariate twin analyses

Twin correlations are included in Table4. For all of the 10 measures, MZ twin correlations exceeded DZ twin correlations indicating the presence of genetic effects. All MZ correlations were less than 1.0 indicating that all measures contained nonshared environmental influences (which also include measurement error). Evidence for shared environmental effects was negligible because DZ twin correlations were less than half the MZ twin correlations on each of the measures (with the exception of neuroticism), thus indicating a possible role for nonadditive genetic influences.

**Table 4:** Twin correlations for FFM traits and SDQ subscales, and variance component estimates

	Twin correlations							Variance components (CIs 95%)			
	MZ	DZ	MZm	MZf	DZm	DZf	DZos	A	D	C	E
Anxiety	.43	.17	.40	.44	.13	.22	.16	.28 (.13–.42)	.13 (.00–.29)	–	.58 (.55–.62)
Conduct	.32	.11	.35	.40	.06	.20	.13	.12 (.00–.27)	.28 (.12–.44)	–	.59 (.56–.64)
Hyperactivity	.44	.16	.44	.44	.15	.10	.15	.07 (.00–.22)	.38 (.22–.49)	–	.55 (.50–.59)
Peer problems	.44	.20	.42	.43	.24	.29	.20	.44 (.32–.48)	.00 (.00–.12)	–	.56 (.53–.59)
Prosociality	.42	.12	.40	.44	.16	.25	.12	.24 (.08–.38)	.18 (.02–.35)	–	.58 (.55–.62)
N	.29	.17	.13	.34	.18	.13	.15	.23 (.06–.32)	–	.04 (.00–.17)	.74 (.67–.79)
E	.39	.15	.38	.39	.15	.14	.12	.14 (.00–.36)	.25 (.00–.44)	–	.61 (.55–.67)
O	.36	.08	.41	.34	.14	.02	.10	.00 (.00–.18)	.37 (.30–.42)	–	.63 (.58–.69)
A	.27	.09	.20	.27	.06	.07	.10	.08 (.00–.27)	.17 (.00–.31)	–	.76 (.69–.81)
C	.37	.05	.28	.39	.04	.07	.09	.00 (.00–.18)	.36 (.30–.41)	–	.64 (.59–.71)

MZ male pairs: *n* = 282–3 (FFM) and 635–6 (SDQ); MZ female pairs: *n* = 501–7 (FFM) and 912–4 (SDQ); DZ male pairs: *n* = 267–271 (FFM) and 580 (SDQ); DZ female pairs: *n* = 396–402 (FFM) and 811–12 (SDQ); and DZ opposite-sex pairs: *n* = 566–568 (FFM) and 1340–43 (SDQ). A = additive genetic effects; C = shared environmental effects; D = dominance effects; E = nonshared environmental effects; MZm = MZ male pairs; MZf = MZ female pairs; DZm = DZ male pairs; DZf = DZ female pairs; DZos = DZ opposite-sex pairs.

Sex differences in the magnitude of genetic and environmental influences on variance were not apparent, despite the large mean sex differences on anxiety and prosociality. For the SDQ scales, the average MZ and DZ correlations were, respectively, .40 and .15 for same-sex male pairs, and .43 and .21 for same-sex female pairs. For FFM traits, the average MZ and DZ correlations were, respectively, .28 and .11 for same-sex male pairs, and .35 and .09 for same-sex female pairs. Qualitative sex differences were also not apparent: Variances (Table1) and twin correlations (Table4) were similar for same-sex and opposite-sex DZ twins. Formal assumption testing, however, yielded significant quantitative (i.e., differences across sex in the magnitudes of variance components) and qualitative (i.e., the extent to which genes and shared environment overlap between males and females) sex effects. However, these effects were modest, in line with the pattern of intraclass correlations, indicating no substantial sex differences. For this reason, and to increase power, we combined males and females for subsequent analyses, with all variables residualized for the mean effect of sex.

We next established the univariate heritability for each of our variables. For variables where DZ correlations were less than twice that of the MZ correlations we modeled dominance effects instead of shared environmental effects (Neale & Cardon, 1992). Accordingly, nonadditive (dominance) effects were modeled for all variables with the exception of neuroticism. Parameter estimates from the univariate modeling are shown in Table4. In brief, model-fitting results confirmed the information provided by the twin correlations, indicating that additive and nonadditive genetic effects were evident for all variables, with no evidence for shared environmental effects (with the exception of neuroticism). The average heritability including additive and nonadditive genetic effects was 0.42 for SDQ traits and 0.32 for FFM traits.

### Multivariate twin analyses

**Figure 1:**
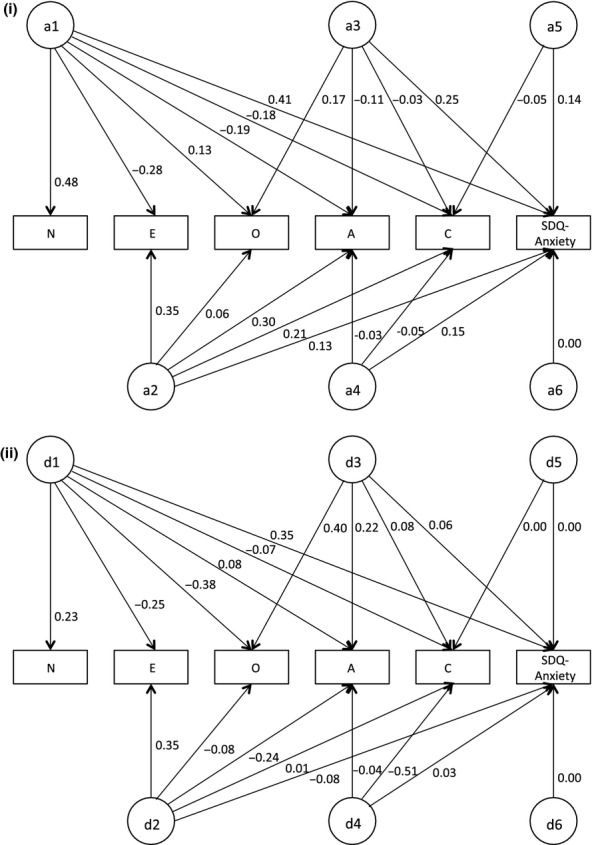
Multivariate (Cholesky) modeling results for additive and dominance genetic effects for FFM traits and SDQ anxiety additive and dominance genetic effects. Note: Additive genetic (a) paths are detailed in the top half of the figure (i), and dominance genetic (d) paths in the bottom half of the figure (ii); six latent factors were modeled for a, d, and e; however, only loadings for a and d are presented above for ease of viewing

We next built a series of multivariate Cholesky models comprised of the FFM traits and one of the SDQ subscales. In each of these models, personality was entered first – in the order N, E, O, A, and C, with one of the SDQ subscales entered last. (Note that the order of the FFM traits is not important in the current analysis as we are examining whether genetic variation in these traits can collectively – rather than individually – explain heritable effects underlying SDQ measures.) This model specifies as many factors as there are variables for each source of variance, with each subsequent factor having one fewer pathways than the preceding factor (see Figure1 and Table5). In other words, for additive genetic effects (a) the first latent factor loads on all of the *n* measured variables: The subsequent latent factors load on *n*−1, *n*−2… *n*−i variables. In this way each factor accounts for as much of the remaining variance as possible, until the last factor accounts for just the remaining variance in the last measured variable, which in this case indicates the genetic variance of the SDQ subscale that is independent of the FFM traits. This is repeated for the dominance genetic factors (d) and nonshared environmental factors (e), with each of these variance components analyzed simultaneously. This design can also be viewed as allowing the genetic and environmental covariation primarily explaining FFM traits to also explain SDQ traits, while also leaving specific genetic and environmental paths available to explain such variation in SDQ traits that does not covary with personality. Because the majority (9 of 10) of our measures showed MZ correlations as greater than twice that of the DZ pairs, we included dominance effects (and excluded shared environmental effects) in each of our multivariate models.

**Table 5:** Multivariate (Cholesky) modeling results for FFM traits and SDQ subscales

	a1	a2	a3	a4	a5	a6	d1	d2	d3	d4	d5	d6	e1	e2	e3	e4	e5	e6
N	.48						.23						.85					
E	−.28	.35					−.25	.35					−.21	.76				
O	.13	.06	.17				−.38	−.08	.40				−.01	.23	.77			
A	−.19	.30	−.11	−.03			.08	−.24	.22	−.04			−.12	.13	.16	.83		
C	−.18	.21	−.03	−.05	−.05		−.07	.01	.08	−.51	.00		−.08	.07	.02	.18	.78	
Anxiety	.41	.13	.25	.15	.14	.00	.35	−.08	.06	.03	.00	.00	.27	−.09	.07	.07	.08	.70
N	.51						−.18						.84					
E	−.30	.35					.22	.36					−.21	.76				
O	.08	.03	.18				.35	−.03	.45				−.01	.23	.77			
A	−.13	.30	−.13	.00			.14	−.33	.04	.02			−.11	.13	.17	.83		
C	−.16	.24	.07	.00	.00		.17	−.08	−.06	.41	.24		−.08	.07	.03	.18	.78	
Conduct	.21	−.11	−.29	.00	.00	.00	−.18	.42	.23	.00	.00	.00	.19	.07	−.03	−.16	−.07	.73
N	.50						.19						.85					
E	−.30	.32					−.23	.37					−.21	.76				
O	.10	.00	.15				−.42	−.04	.39				.00	.23	.76			
A	−.17	.29	−.12	−.06			.05	−.22	.26	−.05			−.12	.13	.17	.83		
C	−.17	.23	.03	.00	.00		−.09	−.01	.05	−.51	.03		−.08	.07	.02	.18	.78	
Hyp.	.20	−.18	−.01	−.10	.00	.00	.24	.42	.27	.24	−.02	.00	.13	.07	.06	−.05	−.19	.70
N	−.51						.15						.85					
E	.27	.32					−.37	−.27					−.21	.75				
O	−.08	−.06	.19				−.44	.22	.28				.00	.23	.77			
A	.16	.30	.02	.14			.06	.25	.19	−.07			−.12	.13	.16	.83		
C	−.17	.20	.10	.05	.00		−.10	.02	.00	−.51	.00		−.08	.07	.02	.18	.78	
Peers	−.30	−.18	.42	.30	.00	.00	.20	−.01	−.11	.01	.00	.00	.18	−.15	.04	−.02	.02	.71
N	.50						.18						.85					
E	−.29	.33					−.27	.36					−.21	.75				
O	.09	.01	.20				−.39	−.07	.39				.00	.23	.77			
A	−.17	.31	−.10	.00			.06	−.22	.24	−.07			−.12	.13	.17	.83		
C	−.18	.24	.09	.00	.00		−.08	−.02	.02	−.50	.00		−.08	.07	.03	.18	.78	
Prosocial	−.29	.32	.22	.00	.00	.00	.22	.17	.31	−.09	.00	.00	−.11	.10	.07	.15	.03	.73

Standardized path coefficients are reported. Hyp = hyperactivity: A = additive genetic effects; D = dominance genetic effects; E = nonshared environmental effects.

The final results of the multivariate modeling are detailed in Table5. The results are illustrated for additive and dominance genetic effects on FFM traits and anxiety in Figure1. Of importance, genetic effects – both additive and dominance – underlying FFM traits were wholly overlapping with genetic influences underpinning each of the SDQ traits. Indeed, no unique genetic influences were observed to account for SDQ measures independent of the FFM traits (i.e., in Table5, a6 and d6 are zero for each of the SDQ subscales). In contrast, nonshared environmental effects showed only a modest overlap between the FFM traits and the SDQ measures, ranging from 9% for prosociality to 17% for anxiety. Genetic and environmental correlations between FFM traits and SDQ measures derived from the multivariate models are detailed in Table6.

**Table 6:** Genetic and environmental correlations between FFM traits and SDQ measures

	A_r_					D_r_					E_r_				
	N	E	O	A	C	N	E	O	A	C	N	E	O	A	C
Anxiety	.76	−.29	.87	−.34	−.44	.96	−.75	−.51	.48	−.20	.35	−.20	.05	.04	.08
Conduct	.56	−.59	−.54	−.17	−.72	.36	.52	.10	−.83	−.29	.24	.02	−.01	−.22	−.14
Hyperactivity	.69	−.93	.34	−.76	−.91	.40	.37	−.03	−.10	−.42	.17	.05	.10	−.05	−.27
Peer problems	.48	−.54	.86	−.23	−.18	.87	−.68	−.93	−.16	−.21	.24	−.26	−.01	−.08	−.02
Prosociality	−.60	.89	.18	.72	.98	.52	.01	.10	.38	.13	−.14	.16	.12	.24	.11

A = Agreeableness; C = Conscientiousness; E = Extraversion; N = Neuroticism; O = Openness; A_r_ = additive genetic correlation; D_r_ = dominance genetic correlation; E_r_ = nonshared environment correlation.

## Discussion

These results confirm work suggesting that adolescent problem behaviors can, in part, be integrated within the normal personality lexicon. Of primary importance, the genetic influences on SDQ measures were entirely shared by genetic influences on FFM traits. These findings, then, at least at the genetic level, are consistent with a model positing that behavior problems are extreme scores on a continuum of otherwise normal personality. FFM traits, however, were not sufficient to explain the total phenotypic variation in SDQ: Between a fifth and a third of the variance in SDQ measures was accounted for by FFM personality traits. This residual variation on SDQ measures was explained by nonshared environmental effects: No evidence was found for shared environmental effects on either SDQ measures or FFM traits.

Our findings also suggest that nonadditive genetic effects are present in both normal personality and problem behaviors. This observation, while often overlooked in the literature (commonly because of power issues; Eaves, Heath, Neale, Hewitt, & Martin, 1998), is consistent both with recent molecular genetic work (Verweij et al., 2012), as well as quantitative genetic research (Eaves et al., 1998; Keller, Coventry, Heath, & Martin, 2005). This finding gives rise to two specific implications concerning the underlying biological bases of both normal personality and problem behaviors. First, genetic association studies may have, in part, failed to reliably locate DNA variants underlying personality and behavior problems (Munafo & Flint, 2011) because such approaches typically use methods designed to detect additive genetic effects. Uncovering genetic mechanisms underpinning personality and behavior problems may require explicit modeling of gene interactions, although this approach, especially within a genome-wide context, is not without its own difficulties (Cordell, 2009). Second, transmission of risk alleles for adolescent behavior problems may be less substantial than commonly implied through estimates of broad-sense heritability: Indeed, only additive genetic effects, reflecting narrow-sense heritable influences, are reliably transmitted to offspring (Plomin et al., 2013).

Of some interest, the additive and dominance genetic correlations between FFM traits and SDQ measures were not always mirrored; that is, whereas some of the additive genetic correlations were positively signed, inverse dominance genetic correlations were noted. For example, we observed that the additive genetic effects underlying openness and anxiety were positively correlated, but inversely correlated for dominance effects. These observations suggest that the shared etiology between personality and adolescent behavioral problems may be complex, even at the genetic level. This particular result for openness and anxiety may also reflect the specific operationalization of openness in the current measures: here, some of the adjectives through which high openness was defined included ‘strange’ and ‘odd’, and so it is possible that the measure tapped into more aberrant forms of openness, as well as the more common aspects (e.g., imaginative).

Specific recommendations for future research arise from the current observations. First, our study used self-report measures; however, some research has noted that results from other report can deviate from self-report (Saudino et al., 2005): Future work should explore this possibility with the present range of measures. Second, the classical twin design (using only MZ and DZ reared-together twins) cannot distinguish between nonadditive genetic effects and shared environmental effects because one masks the other (Neale & Cardon, 1992). While our modeling assumed zero shared environmental effects, with the current design we can only confidently claim that nonadditive genetic effects are larger than shared environmental effects. Future research, then, should seek to use extended twin and family designs to address this possibility as they afford simultaneous estimation of these parameters. Third, while our results indicate a common genetic etiology underpins FFM traits and SDQ measures, these results do not indicate which genes are in common. Nonetheless, although little will advance the field more than finding specific genes that account for some of the heritability of FFM traits or SDQ measures, the present results predict that genes associated with one domain will likely also be associated with the other domain. Fourth, our measure of neuroticism may have shown an inflated association with SDQ measures such as anxiety because of content overlap. Because of the limited number of items in our current measure of neuroticism (six items), reanalyses with a smaller set of items would not have provided an especially useful test of whether significant links remain after such content overlap is removed. Moreover, work directly addressing this issue has reported that meaningful links remain even controlling for apparently contaminating items (Uliaszek et al., 2009). Nonetheless, future work could fruitfully extend existing research on this topic so as to further clarify the relevance of this concern. However, it is noteworthy that the FFM traits share all of the genetic variance with other SDQ measures, which prima facie appear to share less content with the FFM traits. Fifth, while links from conduct to traits broadly reflecting (low) conscientiousness and agreeableness are in line with meta-analytic research (Miller & Lynam, 2001), we also saw arguably less well-observed links with extraversion and neuroticism. These associations may reflect the anger-hostility elements of neuroticism and the sensation-seeking elements of extraversion (e.g., Raine et al., 1998). Future work is recommended to address this possibility. Finally, while genetic effects on FFM traits were wholly overlapping with SDQ measures, the same levels of comorbidity were not observed for nonshared environmental effects. This, in part, might reflect measurement error (nonshared environment effects also contain measurement error and this is not shared between traits), but also suggests that specific environmental factors may influence adolescent behavioral problems. Future work, then, should include measures of the environment so as to identify the sources of these nonshared environmental effects.

In summary, we provide evidence from a large, nationally representative twin sample that behavioral problems in adolescence can be understood within the normal personality lexicon, namely, the five-factor model of personality. Moreover, additive and dominance genetic effects underpinning normal personality traits were wholly overlapping with those genetic effects underlying adolescent behavioral problems.
